# De Garengeot’s hernia: Case report and literature review

**DOI:** 10.1016/j.ijscr.2019.09.037

**Published:** 2019-09-28

**Authors:** Carlos Bustamante Recuenco, Javier García-Quijada García, Manuel Cendrero Martín, Alberto Carabias Hernández, Ana Serantes Gómez, Paloma Sanz Muñoz, Miguel Ángel Delgado Millán, José María Jover Navalón

**Affiliations:** Hospital Universitario de Getafe, Madrid, Carretera Madrid-Toledo km 12.5, 28905, Spain

**Keywords:** CRP, C-reactive protein, CT-scan, computerized tomography scan, SSI, surgical site infection, TEP, totally extraperitoneal hernia repair, TAPP, trans-abdominal pre-peritoneal hernia repair, Hernia, Garengeot, Appendix, Preperitoneal, Diagnosis, Prosthesis

## Abstract

**Introduction:**

De Garengeot’s Hernia is a rare type of femoral hernia in which the appendix is located inside the herniated sac. Diagnosis of the condition is challenging and its treatment must be performed without delay.

**Presentation of case:**

We present the case of a 75-year-old patient with a femoral hernia in which an appendix with signs of inflammation was found. An appendectomy followed by hernia repair was performed under an open preperitoneal approach according to Nyhus technique. The patient did not present any complications and was discharged on the second postoperative day.

**Discussion:**

This type of hernia is often unexpected and its preoperative diagnosis is difficult to perform. In most cases the clinical picture is indistinguishable from a common incarcerated hernia. Contrast-enhanced CT is the most useful complementary test, although it is not as accurate as desirable, so the diagnosis is frequently found intraoperatively. There is a wide variety of surgical options and there is no consensus on the most appropriate one. The preperitoneal approach enables the performance of an appendectomy and subsequent hernia reparation. The use of prosthesis should be considered if there are no signs of perforation or abscess.

**Conclusion:**

De Garengeot’s hernia is a very rare entity. The diagnosis and subsequent surgical treatment must be early to prevent the disease progression. The preperitoneal approach should be considered as the first choice technique, as it allows the exploration of the herniated sac and the performance of surgical procedures on its content.

## Highlights

• De Garengeot’s hernias constitute an extremely rare condition associated with emergency surgery.
• Suspicion and experience are crucial for correct management, as no therapeutic or diagnosis consensus has been reached.
• A single surgical procedure can be performed to treat both pathologies; incarcerated hernia and strangulated appendix.
• An open preperitoneal approach permits correct visualization of the sac contents and permits appendectomy and hernia repair.
• When a complication is found, such as local perforation or abscess, a two-step abdominal wall repair should be considered.

## 1 Introduction

The presence of the appendix within a femoral hernia was first described in 1731 by René Jacques Croissant De Garengeot [1]. It is considered an extremely rare clinical entity, as an incidence of 0.5–5% of all the femoral hernias surgically treated is reported in the literature, and less than 200 cases have been hitherto presented [2]. Its clinical presentation is often indistinguishable from any other type of incarcerated groin hernia. In certain cases infection symptoms can appear due to the presence of acute appendicitis inside the hernia sac [3]. Although its diagnosis can be performed using imaging tests, in most cases it is made intraoperatively. It requires emergency surgery, although there is no consensus on the specific surgical approach. This work has been reported in line with the SCARE criteria [4].

## 2 Presentation of case

Herein, we describe the case of a 75-year-old woman with no relevant medical history, who arrived at our emergency department complaining of right inguinal pain experienced over the last 12 h, and associated with the appearance of a lump at that same level. The patient did not present any intestinal obstruction and was afebrile upon presentation. Physical examination revealed a non-reducible right inguinal mass hard in consistency, painful and without signs of local swelling or warmth.

Leucocytosis with associated neutrophilia was identified. The abdominal X-ray did not show any pathological findings.

Due to the clinical and analytical findings, a diagnosis of incarcerated inguino-femoral hernia was made and the patient was transferred to the operating room.

**Fig. 1:**
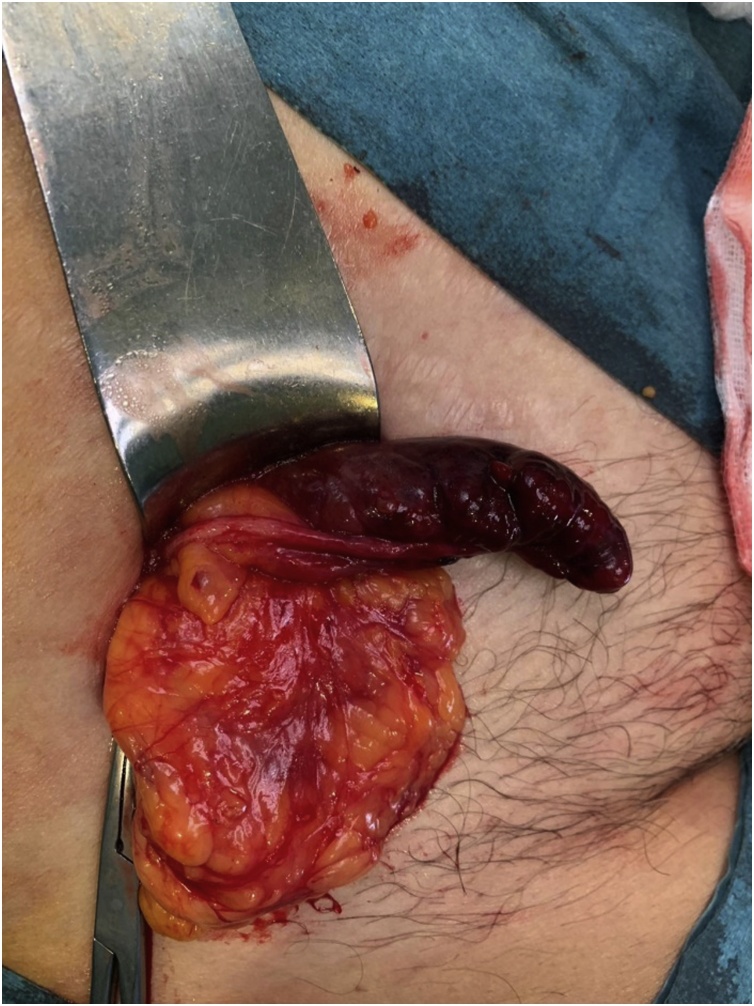
An isquemic appendix without signs of perforation was found inside the hernia sac.

Under general anesthesia, a preperitoneal approach was performed according to the Nyhus technique. An incarcerated femoral hernia was found. The sac was opened, and an isquemic appendix without signs of abscess or perforation was revealed (Fig. 1). The cecum was identified and the appendectomy was performed. Then, the hernia sac was reduced, and a 15 × 15 cm, low density polypropylene mesh was placed at a preperitoneal level and fixed (with prolene suture) to the Cooper ligament.

Oral tolerance was initiated on the first postoperative day and no unexpected events ocurred. The patient was discharged 48 h after the operation in good general condition.

The anatomo-pathological analysis reported an appendix with signs of ischemia.

## 3 Discussion

Femoral hernias constitute an uncommon subtype of groin hernias, representing around 3% of all abdominal wall hernias, and usually appear in elder women [5]. Because of the narrow wall defect, femoral hernias have an increased risk of incarceration. The presence of the appendix as a content of the herniated sac is very uncommon, as it has an estimated frequency of 0.5–5% out of all femoral hernias, although its true incidence is difficult to establish. The presence of a mobile cecum or certain grade of bowel malrotation act as predisposing factors [3,6]. The finding of a perforated appendix is even rarer, and it is explained by the ischemic mechanism produced by the incarceration process [7].

The most frequent clinical scenario is the appearance of a groin mass (usually right) associated with abdominal pain. The presence of fever is found in 8–10% of the patients, and it is not related to the severity or the prognosis of the disease. Physical findings include abdominal pain, non reductable inguinal mass and local signs of inflammation (erythema, warmth, tenderness) in one third of the cases [3]. Obstructive symptoms are uncommon, as the femoral canal’s diameter often doesn’t allow for the incarceration of the appendix and a bowel segment at the same time [8]. Occasionally, there is absence of groin symptoms, a fact that makes the diagnosis more challenging and delays surgical treatment.

Our patient showed a typical case of complicated hernia, as she presented an abdominal pain associated with the recent appearance of a right groin mass.

Due to the lack of reports in literature and its low incidence, there’s not a management protocol to guide the diagnostic and treatment process in this pathology. In fact, a recent meta-analysis [3] didn’t establish any recommendations in this regard.

An effective preoperative diagnosis is often hard to achieve, and will demand laboratory and imaging tests. An increase in CR-Protein or white blood cell count can be present in half of the patients, although it is not associated with the severity or outcome of the disease. Among the available imaging tests, the abdominal CT-scan is the most sensitive and specific, in spite of the report of a 40% sensibility and an accuracy of about 60–70% [9,10]. One of the causes of its low efficacy is the misinterpretation of the herniated content, conditioned by the lack of expertise of the radiologist in these cases [11,12]. This misjudgement of the results, added to the delay in treatment associated with performing these tests, raises skepticism regarding the usefulness and whether they should be routinely required. In our opinion, performing a CT scan as a standardized process in all incarcerated hernia cases is not justified. We believe that surgical exploration of the herniated content is technically feasible and will reveal the presence of the appendix without any delays in treatment stemming from unnecessary imaging tests.

As mentioned above, there is not an updated recommendation about the surgical approach to be carried out on these patients. In fact, the previously reported meta-analysis concludes that the surgical approach should be determined by each surgeon depending on his individual experience and the specific characteristics of the case [3]. Among all options, the anterior approach is the most commonly employed, despite the higher rate of additional laparotomy performed due to the difficulty to access the appendix base and explore the abdominal cavity [13]. McBurney’s incision and sub-umbilical midline laparotomy are the most common additional incisions [6], being the latter the chosen approach when there exist reasonable risk of complications such as bowel obstruction or incarcerated appendicitis with local perforation or abscess [14].

Laparoscopic approach has emerged as an alternative in this scenario. It has evident advantages, such as the possibility of a complete abdominal exploration and the performance of easy procedures as appendectomy [14]. It also permits abdominal wall reparation via TEP or TAPP.

In our opinion, although the laparoscopic approach offers some clear advantages, we believe that it should not be go-to choice in complicated hernias. Only experienced surgeons capable of performing both laparoscopic intestinal resection and hernioplasty (uncommon fact at the present) should consider this approach, as more inexperienced practitioners could be unable to deal with the potential risks that could emerge. In other instances, additional laparotomy will be necessary to achieve a correct surgical treatment. Even in case of a De Garengeot’s hernia correctly diagnosed preoperatively and subsequent laparoscopic appendectomy successfully completed, a conversion will be necessary to perform a hernioplasty if a trained surgeon in this technique is not present.

For the disadvantages associated with previous mentioned techniques, we believe that the open preperitoneal approach should be the first option to consider in a De Garengeot’s or in any other kind of complicated hernia. It allows an adequate exploration of the hernia sac and rapid action over its contents. Moreover, it permits the performance of a simple hernioplasty through a single incision. Besides, it does not require a high level technical skill and much experience to be executed correctly.

Abdominal repair with or without prosthetic material is controversial, as the risk of mesh infection can cause great morbidity. Thus, up to two thirds of the surgeons perform anatomic repairs in these patients [15]. It is clear that the use of prosthetic mesh is safe in the absence of local complications, such as abscess and local perforation. However, if these or other risk factors for surgical site infection are present, an anatomic repair or an appendectomy followed by interval hernioplasty must be considered [5]. In the event of complications, the laparoscopic approach is a valid option, as it makes the isolation of the mesh from the infected site feasible, decreasing the risk of surgical site infection. However, there is not solid evidence supporting this at the present [16].

For our patient, we decided on the open preperitoneal approach, which allowed us to perform a conventional open appendectomy followed by preperitoneal hernioplasty with prosthetic mesh in a quick and effective way.

## 4 Conclusion

De Garengeot’s hernia is an uncommon subtype of femoral hernia which can result in high morbidity if incorrectly managed. Preoperative diagnosis is often challenging and correct identification prior to surgery always necessitates imaging tests, among which the abdominal CT-scan shows the best results. Prompt surgical treatment should be performed to avoid further complications. In our opinion, the preperitoneal approach should be considered as the technique of choice for these patients. It enables a correct visualization of the hernia sac contents and simplifies the performance of surgical procedures such as intestinal resection or appendectomy. The placement of prosthetic material can be considered if there are no signs of abscess or local perforation. In any case, there currently exist a wide variety of surgical options for this particular entity, all of which have comparable results and a low rate of postoperative morbidity. For this reason, the surgeon should feel free to choose the surgical approach for each case, based on the pre- and intraoperative findings and his own experience.

## Funding

The authors received no financial support for the research, authorship, and/or publication of this article.

## Ethical approval

Exception from ethical approval-case report only, consent from the patient provided at request.

## Consent

Written informed consent was obtained from the patient for publication of this case report and accompanying images. A copy of the written consent is available for review by the Editor-in-Chief of this journal on request.

## Author contribution

Dr. Bustamante Recuenco C.: study desing. Data analysis and interpretation, writing and submission of the paper.

Sr. García-Quijada García J.: study desing. Data analysis and interpretation, writing and submission of the paper.

Sr. Cendrero Martín M.: data analysis and interpretation, writing the paper.

Sr. Serantes Gómez A.: interpretation of data.

Sr. Sanz Muñoz P.: interpretation of data.

Dr. Carabias Hernández A.: interpretation of data, writing the paper.

Dr. Delgado Millán MA.: interpretation of data.

Dr. Jover Navalon JM.: interpretation of data and submission of the paper.

## Registration of research studies

NA.

## Guarantor

Carabias Hernández A.

Bustamante Recuenco C.

García-Quijada García J.

Jover Navalon JM.

## Provenance and peer review

Not commissioned, externally peer-reviewed.

## Declaration of Competing Interest

The authors declare no conflict of interest.
